# Mammalian Target of Rapamycin Signaling Pathway Regulates Mitochondrial Quality Control of Brown Adipocytes in Mice

**DOI:** 10.3389/fphys.2021.638352

**Published:** 2021-07-14

**Authors:** Bahetiyaer Huwatibieke, Wenzhen Yin, Lingchao Liu, Yuxin Jin, Xinxin Xiang, Jingyan Han, Weizhen Zhang, Yin Li

**Affiliations:** ^1^Department of Physiology and Pathophysiology, School of Basic Medical Sciences, Peking University, Beijing, China; ^2^Department of Integration of Chinese and Western Medicine, Tasly Microcirculation Research Center, Peking University Health Science Center, Beijing, China; ^3^Department of Pathology, Central Hospital of Zibo, Zibo, China

**Keywords:** brown adipose tissue, mitochondria, mammalian target of rapamycin, obesity, white adipose tissue

## Abstract

The mammalian target of rapamycin (mTOR) is an important protein kinase that senses changes in extracellular and intracellular energy levels and plays a key role in regulating energy metabolism. Brown adipose tissue, which can be converted to white adipose tissue, contains a large number of mitochondria and regulates energy expenditure through thermogenesis. Because obesity is a process of fat accumulation due to chronic excessive energy intake, we attempted to determine whether the mTOR signaling pathway can affect the mitochondrial quality control of brown adipocytes through sensing energy status, thereby regulating brown/white adipocyte transformation. In the present study, through activation or inhibition of mTOR signaling, we detected mitochondrial biogenesis, dynamics, and autophagy-related markers in brown adipocytes. We found that activation of mTOR signaling downregulated the expression of mitochondrial biogenesis, dynamics, and autophagy-relevant markers and inhibited the mitochondrial quality control of brown adipocytes, indicating a phenotypic transformation of brown to white adipocytes. In contrast, inhibition of mTOR signaling upregulated the expression of mitochondrial biogenesis, dynamics, and mitophagy-relevant markers and strengthened mitochondrial quality control, suggesting an inhibition of the phenotypic transformation of brown to white adipocytes. In conclusion, the mTOR signaling pathway plays an important role in modulating the transformation of adipocytes by regulating mitochondrial quality control.

## Introduction

Obesity results from a fat accumulation process caused by long-term excess energy intake, which is closely related to the development of “metabolic syndrome,” such as diabetes, hypertension, hyperlipidemia, and atherosclerosis (Kopelman, 2000; Stacchiotti et al., 2014). The incidence of obesity has increased year by year; in addition to reducing energy intake, increasing energy consumption is also an important way in treating obesity.

Adipose tissue is generally divided into two types, white and brown (Soni et al., 2019). White adipose tissue stores excess energy in the form of large monolocular lipid droplets (Cinti, 2005), while brown adipose tissue is rich in mitochondria, with small multilocular lipid droplets (Soni et al., 2019). The uncoupling protein 1 (UCP1) on the mitochondrial membrane of brown adipocytes transports protons across the mitochondrial membrane to stop oxidative phosphorylation, causing more than 50% of energy used in the synthesis of ATP to be released in the form of heat (Cinti, 2002; Cannon and Nedergaard, 2004). Therefore, brown adipose tissue accelerates energy metabolism and promotes fat consumption. Adipose tissue dysfunction is a central mechanism of obesity and related metabolic diseases (Longo et al., 2019). Brown adipocytes are able to dissipate energy in the form of heat (thermogenesis) and therefore exhibit antiobesity and antidiabetic properties. Active brown adipose tissue exists in humans, which can be activated by cold and is negatively associated with obesity. Promotion of brown adipose tissue (BAT) activity or browning of white adipose tissue (WAT) is associated with cold tolerance *in vivo*, increased energy expenditure, and protection against obesity (Fenzl and Kiefer, 2014).

Mammalian targets of rapamycin (mTOR) are highly conserved serine–threonine protein kinases involved in the regulation of protein transcription, translation, and control of cell growth by sensing intracellular and extracellular energy status in the form of phosphorylation activation (Vila-Bedmar et al., 2010; Yin et al., 2014). mTOR is closely related to obesity and plays a vital role in regulation of energy metabolism. The mTOR complex 1 (mTORC1-) and mTORC2-related signaling pathways play multiple important roles in brown and beige adipocytes and thermogenesis. The current study suggests that mTORC1- and mTORC2-related signaling is involved in thermogenesis through regulation of lipid metabolism (lipolysis and lipogenesis), thermogenic gene expression, and mitochondrial biogenesis and function (Ye et al., 2019). In adipose tissue, mTORC1 activation promotes adipogenesis by activating PPAR-γ, and mTORC2-Akt activation reduces lipolysis and promotes glucose uptake. High circulating nutrients and cytokines in obesity promote mTORC1 activity, which inhibits insulin signaling and causes insulin resistance (IR) through multiple mechanisms (Laplante and Sabatini, 2012). Although studies have shown that mTOR can promote adipogenic differentiation of stem cells, few studies have shown a role for mTOR in BAT. It has been shown that during the development of BAT, mTOR phosphorylation levels show a time-dependent decrease, and knockdown of the mTORC1 component Raptor enhances adipocytes’ mitochondrial function (Inoki et al., 2005; Polak et al., 2008). Therefore, mTOR may affect mitochondrial function and be involved in the phenotypic transformation of brown/white adipocytes.

Mitochondria, which produce at least 90% of the heat in humans, have self-replication, transcription, and coding functions (Chance et al., 1979). Each cell contains mitochondria, but the number of mitochondria in various cells is different (Robin and Wong, 1988). Mitochondrial quality control mainly includes four aspects: mitochondrial biogenesis, dynamics, mitophagy, and the mitochondrial permeability transition pore (MPTP) (Yan et al., 2012; Stacchiotti et al., 2020). Mitochondrial biosynthesis is mainly regulated by the AMPK-PGC1α pathway and Sirt1-PGC1α pathway. AMPK induces phosphorylation of PGC1α whereas Sirt1 stimulates deacetylation of PGC1α and then induces mitochondrial biogenesis. In the nucleus, PGC1α activates nuclear respiratory factor NRF1 and NRF2 through protein–protein interaction and then activates mitochondrial transcription factor A (Tfam) in regulating the biogenesis of mitochondria (Li et al., 2017). Mitochondrial dynamics include fusion and fission. Mitochondrial fusion is mainly regulated by Mitofusin1 (Mfn1), Mitofusin2 (Mfn2), and optic atrophy 1 (OPA1), whereas mitochondrial fission is mainly regulated by mitochondrial fission factor (MFF), Fission1 (Fis1), and dynamin-related protein 1 (Drp1) (Westermann, 2010; Stacchiotti et al., 2014). Autophagy-related genes (Atgs) regulate autophagy, and BNIP3 can specifically regulate mitochondrial autophagy (Romanello et al., 2010). Mitochondrial abnormalities (defective mitochondrial biogenesis and impaired mitochondrial dynamics) and mitophagy defects are major cellular events that occur during the genesis, development, and pathogenesis of Alzheimer’s disease, and in particular, mitophagy defects affect the expression of proteins associated with mitophagy (PINK1, BNIP3, and LC3-1) as well as autophagy-associated proteins (ATG proteins) (Oliver and Reddy, 2019; Reddy and Oliver, 2019; Pradeepkiran and Reddy, 2020; Tran and Reddy, 2020).

Previous studies have shown that activation of mTORC1 signaling stimulates the conversion of brown adipocytes to white adipocytes (Xiang et al., 2015). Because obesity is caused by excessive energy intake over time, can the energy sensor mTOR affect the mitochondrial quality control of brown adipocytes and thus regulate the “whitening” of brown adipocytes? Therefore, we propose the following hypothesis: the mTOR signaling pathway can regulate brown adipocytes’ “whitening” by affecting mitochondrial quality control. In this study, we investigated the role of the mTOR signaling pathway in mitochondrial quality control of brown adipocytes and found that mTOR activation inhibits mitochondrial biogenesis, mitochondrial dynamics, and mitochondrial autophagy of brown adipocytes, thus affecting the phenotypic transformation of brown/white adipocytes.

## Materials and Methods

### Ethics Statement

The animals used in this study were handled in accordance with the Guide for the Care and Use of Laboratory Animals published by the United States National Institutes of Health (NIH publication no. 85–23, revised 1996), and all the experimental protocols were approved by the Animal Care and Use Committee of Peking University. The investigators understand the ethical principles under which this journal operates and confirm that the work complies with the journal animal ethics checklist.

### Materials

Phospho-S6 (ser235/236) and S6 rabbit monoclonal antibodies, Phospho-mTOR (ser2448) and mTOR rabbit monoclonal antibodies, and UCP1 rabbit monoclonal antibody were purchased from Cell Signaling Technology (Beverly, MA, United States). Mouse anti-β-actin was purchased from Santa Cruz Biotechnology (Santa Cruz, CA, United States). PGC1α rabbit monoclonal antibody, Mfn2, OPA1, and Drp1 rabbit monoclonal antibody were purchased from Abcam (Cambridge, United Kingdom). Insulin, indomethacin, isobutylmethylxanthine, dexamethasone, leucine, and collagenase I were from Sigma-Aldrich (St. Louis, MO, United States). IRDye-conjugated affinity purified anti-rabbit and anti-mouse IgGs were purchased from Rockland (Gilbertsville, PA, United States). TRIzol reagent and the reverse transcription (RT) system were purchased from Invitrogen Inc. (Grand Island, NY, United States).

### Animals and Animal Care

Tsc1^*loxp/loxp*^ mice in which exons 17 and 18 of the Tsc1 gene and mTOR^*loxp/loxp*^ mice in which exons 1–5 of the mTOR gene are flanked by loxP sites by homologous recombination were purchased from The Jackson Laboratory (Bar Harbor, ME, United States). Neonatal in-house-bred C57BL/6J, Tsc1^*loxp/loxp*^, and mTOR^*loxp/loxp*^ mice were used in this study. Parental animals were housed under a 12–12-h light–dark cycle at 21°C and had *ad libitum* access to food and water. Regular chow and water were available *ad libitum*.

### Culture of Primary Brown Adipocytes

Brown pre-adipocytes were isolated by digesting the interscapular BAT of neonatal mice with collagenase and mechanistic dispersion, as described previously (Wu et al., 2012). Isolated cells were randomly plated in tissue-culture dishes in DMEM supplemented with 20% FBS. After 24 h culture at 37°C, cells were rinsed twice with phosphate-buffered saline (PBS), after which 70% of the initial cells were attached to the dish, forming a monolayer. Isolated brown pre-adipocytes were cultured in DMEM supplemented with 20% FBS for proliferation. To induce brown adipocyte differentiation, cells were cultured for 2 days in 10% FBS-DMEM supplemented with 20 nmol/l insulin, 1 nmol/l T3, 12.5 mmol/l indomethacin, 0.5 mmol/l isobutylmethylxanthine, and 2 mg/ml dexamethasone. On days 3–6, the induction medium was substituted by a maintenance medium consisting of DMEM supplemented with 20 nmol/l of insulin and 1 nmol/l of T3. For the undifferentiated controls, brown pre-adipocytes were cultured in 10% FBS-DMEM without other supplements. During differentiation, leucine and/or rapamycin were added to the differentiation medium once every 2 days, while saline and/or DMSO was added to the control group. Cell culture was done at least four times for each condition.

### Adenovirus Infection

The Cre adenoviruses were expanded, titrated in 293 cells, and purified by cesium chloride methods as described previously (Wang et al., 2002). For adenovirus-mediated gene transfer, brown adipocytes were infected with 10^6^ titer adenovirus for 48 h. Infection efficiency was judged by green fluorescent protein (GFP) expression observed under the microscope (Leica Microsystems GmbH, Wetzlar, Germany). Infected primary brown pre-adipocytes were differentiated for 6 days and then harvested for subsequent analysis.

### RNA Extraction and Quantitative Real-Time PCR Analysis

Total RNA was isolated using the TRIzol reagent. Reverse transcription was performed using the RT system according to the manufacturer’s instructions. PCR was conducted in a 25-μl volume containing 2.5 μl cDNA, 5 mM MgCl_2_, 0.2 mM dNTPs, 0.2 μM of each primer, 1.25 U AmpliTaq polymerase, and 1 μl 800 × diluted SYBR Green I stock using the M × 3,000 multiple quantitative PCR system (Strata gene, La Jolla, CA, United States). PCR reactions were performed in duplicate, and each experiment was repeated six times. Primers used in this study are shown in Table 1. The mRNA expression was quantified using the comparative cross threshold (CT) method. The CT value of the housekeeping gene β-actin was subtracted from the CT value of the target gene to obtain Δ CT. The normalized fold changes of target gene mRNA expression were expressed as 2^-ΔΔ^CT^^, where ΔΔ CT equals to Δ CT sample-Δ CT control.

**TABLE 1:** Required primer sequences.

	**Upstream primer**	**Downstream primer**
Mouse UCP1	GGACGACCCCTAATCTAATG	CATTAGATTAGGGGTCGTCC
Mouse UCP2	GGAGAGTCAAGGGCTAGT	ACTAGCCCTTGACTCTCC
Mouse UCP3	ATCAGGATTCTGGCAGGC	GCCTGCCAGAATCCTGAT
Mouse PGC1α	GATTGAAGTGGTGTAGCGAC	GTCGCTACACCACTTCAATC
Mouse NRF1	TCTGGTACATGCTCACAGGG	ACTCTGGAGGAAGCCACCTT
Mouse NRF2	TGCCTCCAAAGGATGTCAAT	CCTCTGCTGCAAGTAGCCTC
Mouse Tfam	TCTGCTCTTCCCAAGACTTCA	GCAAAGGATGATTCGGCTC
Mouse COX4	GCCCCATCCCTCATACTTTC	GCTCTCACTTCTTCCACTCATTCT
Mouse COX8B	GAACCATGAAGCCAACGACT	GCGAAGTTCACAGTGGTTCC
Mouse ATP5B	GACATGGGCACAATGCAGG	GCAGGGTCAGTCAGGTCATCA
Mouse ELOVL3	CGTAGTCAGATTCTGGTCCT	CCAGAAGAAGTGTTCCGTTG
Mouse Mfn1	ATTGGGAGGTGCTGTCTC	TTCGGTCATAAGGTAGGCTTT
Mouse Mfn2	AGATGTCCCTGCTCTTTTCTC	TGTGTTCCTGTGGGTGTCTT
Mouse OPA1	TCTGAGGCCCTTCTCTTGTT	TCTGACACCTTCCTGTAATGCT
Mouse Drp1	CGGTTCCCTAAACTTCACGA	GCACCATTTCATTTGTCACG
Mouse Fis1	AAGTATGTGCGAGGGCTGTT	GGCAGAGAGCAGGTGAGG
Mouse Beclin	CTCCATTACTTACCACAGCCCA	AAATGGCTCCTCTCCTGAGT
Mouse Atg7	TGACCTTCGCGGACCTAAAG	AGGGCCTGGATCTGTTTTGG
Mouse BNIP3	GAAGCGCACAGCTACTCTCA	TCCAATGTAGATCCCCAAGCC
Mouse GAPDH	ATGACATCAAGAAGGTGGTG	CATACCAGGAAATGAGCTTG
Mouse β-actin	ATCTGGCACCACACCTTC	AGCCAGGTCCAGACGCA

### Western Blot Analysis

Cultured cells were harvested and homogenized in ice-cold fractionation buffer containing RIPA lysis buffer, phenylmethanesulfonyl fluoride (PMSF), and protein phosphatase inhibitor mixture. The cell lysate was treated with ultrasound for 3 s three times, then centrifuged at 12,000 rpm for 10 min at 4°C. After centrifugation, the supernatant was used for western blot analysis. The protein concentration was measured by Bradford’s method. A total of 40–60 μg protein from each sample was loaded. Proteins were transferred to polyvinylidene fluoride membranes. The membranes were incubated for 1 h at room temperature with 4% fat-free milk in Tris-buffered saline containing Tween-20, followed by incubation overnight at 4°C with the individual primary antibodies (PGC1α, UCP1, mTOR, p-mTOR, S6, p-S6, Mfn2, OPA1, and Drp1 diluted 1:1,000). A specific reaction was detected using the IRDye-conjugated second antibody (antibodies were diluted 1:15,000) and visualized using the Odyssey infrared imaging system (LI-COR Biosciences, Lincoln, NE, United States). Quantification of image density in pixels was performed using the Odyssey infrared imaging system (LI-COR Biosciences). Each experiment was repeated at least four times.

### Oil Red O Staining

Brown adipocytes were rinsed in PBS three times, then fixed with 4% paraformaldehyde for 15 min. After washing, samples were incubated in 0.3% oil red staining solution for 45–60 min at room temperature. Samples were then counterstained with hematoxylin for 30 s, followed by washing in PBS for 5 min.

### Statistical Analysis

Data were expressed as means ± SD. Data analysis used GraphPad Prism software. The unpaired Student’s *t*-test (between two groups) was used as appropriate. *P*-values < 0.05 denote statistical significance.

## Results

### Activation of mTOR Inhibits Mitochondrial Biogenesis of Brown Adipocytes

First, to demonstrate the effective differentiation of brown adipocytes in our experiment, brown pre-adipocytes were induced to differentiate for 4–6 days. As shown in Supplementary Figures 1A,B, mRNA levels of brown adipocyte gene markers were significantly increased after induced differentiation. The PGC1α and UCP1 protein expression levels were also increased significantly (Supplementary Figure 1C). Multilocular lipid droplets were detected by oil red O staining (Supplementary Figure 1D), indicating the effective differentiation of brown adipocytes.

**FIGURE 1:**
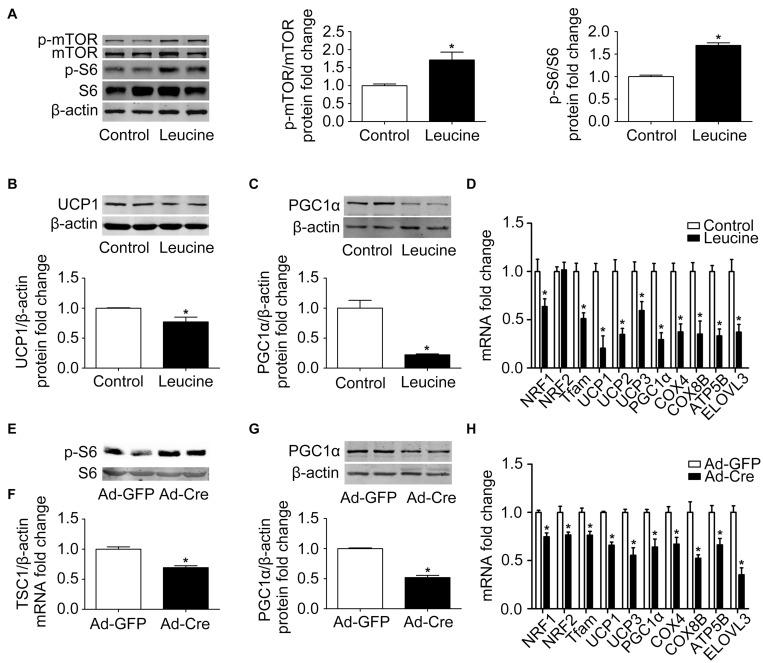
Activation of mTOR inhibits mitochondrial biogenesis of brown adipocytes. **(A–C)** Primary brown adipocytes were isolated from neonatal C57BL/6J mice and cultured for differentiation. Proteins were extracted after 6 days of differentiation. Western blotting of p-mTOR, p-S6, UCP1, and PGC1α were performed with β-actin used as a loading control. Relative protein signal intensity was quantified and expressed as means ± SD. **(D)** Total RNAs were extracted after 6 days of differentiation. Real-time PCR was performed to evaluate the expression of mitochondrial biogenesis marker genes with β-actin used as a loading control and results expressed as means ± SD. *Denotes *P* < 0.05 relative to control. **(E,F)** Tsc1^*loxp/loxp*^ brown adipocytes were treated with Cre adenoviruses for 48 h and controls with GFP adenoviruses. RNAs and protein were extracted as described in the methods section. Real-time PCR was performed to evaluate the expression of TSC1. Levels of mRNA expression were normalized to β-actin and expressed as means ± SD. Western blotting of p-S6 and total S6 was performed. **(G)** Representative results of western blot for PGC1α. β-Actin was used as the loading control. Relative protein signal intensity was quantified and expressed as means ± SD. **(H)** Real-time PCR was performed to evaluate the expression of genes related to mitochondrial biogenesis. Levels of mRNA expression were normalized to β-actin and expressed as means ± SD. *P < 0.05 vs. Ad-GFP.

In order to study the effects of the mTOR signaling pathway on brown adipose mitochondria, we first activated mTOR by chemical treatment. Brown pre-adipocytes were isolated from wild-type (WT) mice and differentiated for 6 days with mTOR agonist leucine processing (15 mmol/l). As shown in Figure 1A, after leucine treatment, the phosphorylation levels of mTOR and its downstream S6 were induced significantly, indicating that the mTOR signaling pathway was successfully activated. Mitochondrial biogenesis-related marker UCP1 and PGC1α protein expression levels were significantly reduced (Figures 1B,C). The mRNA expression levels of mitochondrial biogenesis-related markers, such as NRF1, NRF2, Tfam, UCP1, UCP2, UCP3, PGC1α, COX4, COX8B, ATP5B, and ELOVL3, were also significantly reduced (Figure 1D).

To further validate the results, brown pre-adipocytes were isolated from Tsc1^*loxp/loxp*^ transgenic mice and treated with Cre adenovirus to knock down the tuberous sclerosis complex 1 (Tsc1) gene, the upstream inhibitor of mTOR signaling. As shown in Figures 1E,F, the mRNA expression level of TSC1 was significantly reduced compared with the control Ad-GFP group, and the mTOR downstream signal S6 showed significant phosphorylation, indicating the activation of the mTOR signaling pathway in these cells. The mitochondrial biogenesis-related marker PGC1α protein expression level was significantly reduced (Figure 1G). The mRNA expression levels of mitochondrial biogenesis-related markers, such as NRF1, NRF2, Tfam, UCP1, UCP3, PGC1α, COX4, COX8B, ATP5B, and ELOVL3, were also significantly reduced (Figure 1H). These results suggest that activation of mTOR inhibits the mitochondrial biogenesis of brown adipocytes.

### Inhibition of mTOR Promotes Mitochondrial Biogenesis of Brown Adipocytes

**FIGURE 2:**
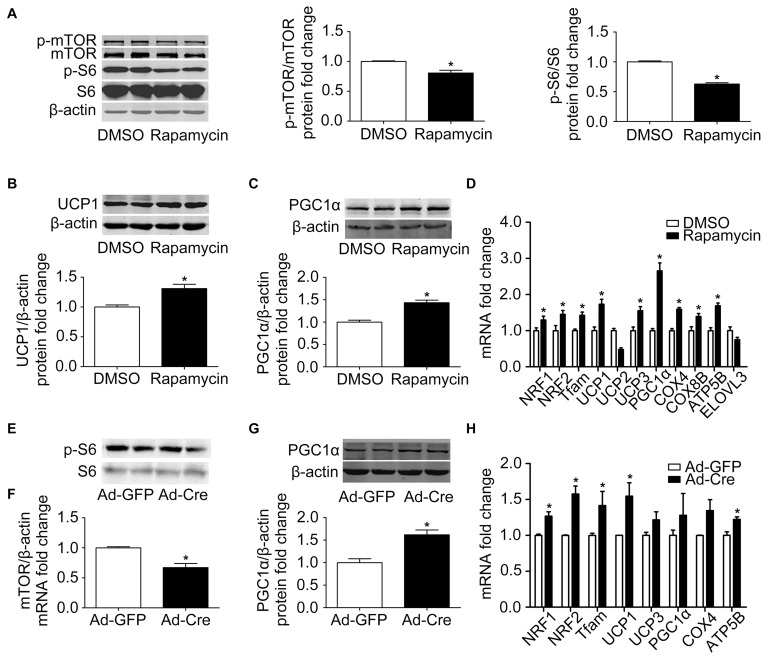
Inhibition of mTOR promotes mitochondrial biogenesis of brown adipocytes. **(A–C)** Primary brown adipocytes were isolated from neonatal C57BL/6J mice and cultured for differentiation. Proteins were extracted after 6 days of differentiation. Western blotting of p-mTOR, p-S6, UCP1, and PGC1α was performed with β-actin as a loading control. Relative protein signal intensity was quantified and expressed as means ± SD. **(D)** Total RNAs were extracted after 6 days of differentiation. Real-time PCR was performed to evaluate the expression of mitochondrial biogenesis marker genes with β-actin as the loading control, with results expressed as means ± SD. *Denotes *P* < 0.05 relative to control. **(E,F)** mTOR ^*loxp/loxp*^ brown adipocytes were treated with Cre adenoviruses for 48 h and controls with GFP adenoviruses. RNAs and protein were extracted as described in the methods section. Real-time PCR was performed to evaluate the expression of mTOR. Levels of mRNA expression were normalized to β-actin and expressed as means ± SD. **(G)** Representative results of western blot for PGC1α with β-actin as the loading control. Relative protein signal intensity was quantified and expressed as means ± SD. **(H)** Real-time PCR was performed to evaluate the expression of genes related to mitochondrial biogenesis. Levels of mRNA expression were normalized to β-actin and expressed as means ± SD. *P < 0.05 vs. Ad-GFP.

In order to further clarify the specific function of the mTOR signaling pathway on brown adipose mitochondria, we inhibited the mTOR signaling pathway. Brown pre-adipocytes were isolated from WT mice and differentiated for 6 days, and at the same time the mTOR inhibitor rapamycin (1 nmol/l) was given. As shown in Figure 2A, after rapamycin treatment, the protein phosphorylation levels of mTOR and its downstream S6 decreased significantly, indicating that mTOR was successfully inhibited. Mitochondrial biogenesis-related marker UCP1 and PGC1α protein expression levels were significantly increased (Figures 2B,C). The mRNA expression levels of mitochondrial biogenesis-related markers, such as NRF1, NRF2, Tfam, UCP1, UCP3, PGC1α, COX4, COX8B, and ATP5B, were also significantly increased (Figure 2D).

To further validate the results, brown pre-adipocytes were isolated from mTOR^*loxp/loxp*^ transgenic mice and treated with Cre adenovirus to knock down the mTOR gene. As shown in Figures 2E,F, the mRNA expression level of mTOR was significantly reduced relative to the control Ad-GFP group, as was the phosphorylation level of mTOR downstream signal S6, indicating the inhibition of the mTOR signaling pathway in these cells. The mitochondrial biogenesis-related marker PGC1α protein expression level was significantly increased (Figure 2G). The mRNA expression levels of mitochondrial biogenesis-related markers, such as NRF1, NRF2, Tfam, UCP1, and ATP5B, were also significantly increased (Figure 2H). These results suggest that inhibition of mTOR promotes the mitochondrial biogenesis of brown adipocytes.

### Activation of mTOR Inhibits Mitochondrial Dynamics of Brown Adipocytes

**FIGURE 3:**
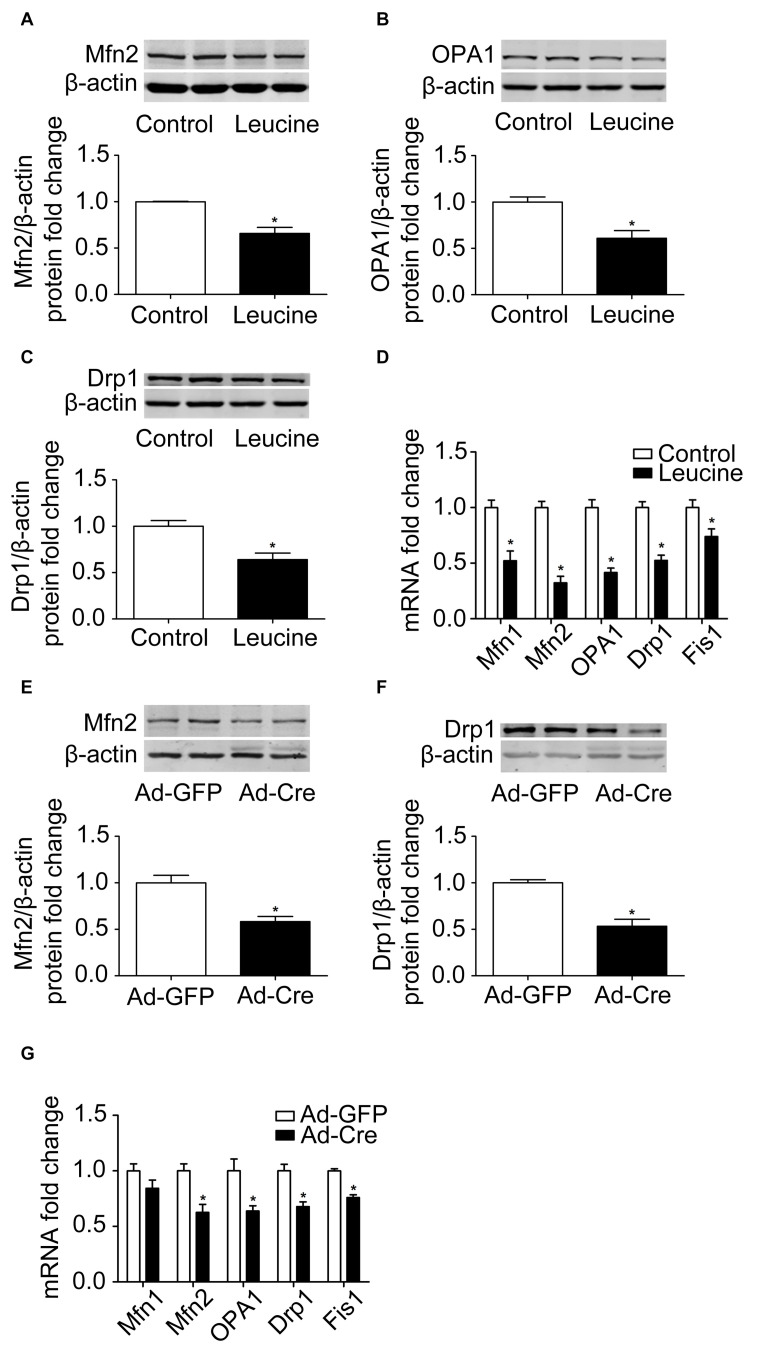
Activation of mTOR inhibits mitochondrial dynamics of brown adipocytes. **(A–C)** Primary brown adipocytes were isolated from neonatal C57BL/6J mice and cultured for differentiation. Proteins were extracted after 6 days of differentiation. Western blotting of Mfn2, OPA1, and Drp1 was performed with β-actin as a loading control. Relative protein signal intensity was quantified and expressed as means ± SD. **(D)** Total RNAs were extracted after 6 days of differentiation. Real-time PCR was performed to evaluate the expression of mitochondrial dynamics marker genes with β-actin as a loading control and results expressed as means ± SD. *Denotes *P* < 0.05 relative to control. **(E,F)** Tsc1^*loxp/loxp*^ brown adipocytes were treated with Cre adenoviruses for 48 h and controls with GFP adenoviruses. RNAs and protein were extracted as described in the methods section. Representative results of western blot for p-S6, S6, Mfn2, and Drp1 are shown. β-Actin was used as the loading control. Relative protein signal intensity was quantified and expressed as means ± SD. **(G)** Real-time PCR was performed to evaluate the expression of genes related to mitochondrial dynamics. Levels of mRNA expression were normalized to β-actin and expressed as means ± SD. *P < 0.05 *vs.* Ad-GFP.

The results above confirmed that the mTOR signaling pathway regulates mitochondrial biogenesis of brown adipocytes. Therefore, we further examined the mitochondrial dynamics. After leucine treatment, mitochondrial fusion-related marker Mfn2 and OPA1 protein expression levels were significantly reduced (Figures 3A,B). Mitochondrial fission-related marker Drp1 protein expression level was also significantly reduced (Figure 3C) as were the mRNA expression levels of mitochondrial dynamics-related markers (Figure 3D).

These results were also verified in TSC1 knockdown cells. As shown in Figures 3E,F, mitochondrial dynamics-related markers Mfn2 and Drp1 protein expression levels were significantly reduced. The mRNA expression levels of mitochondrial dynamics-related markers, such as Mfn2, OPA1, Drp1, and Fis1, were also significantly reduced (Figure 3G). These results suggest that activation of mTOR inhibits the mitochondrial dynamics of brown adipocytes.

### Inhibition of mTOR Promotes Mitochondrial Dynamics of Brown Adipocytes

**FIGURE 4:**
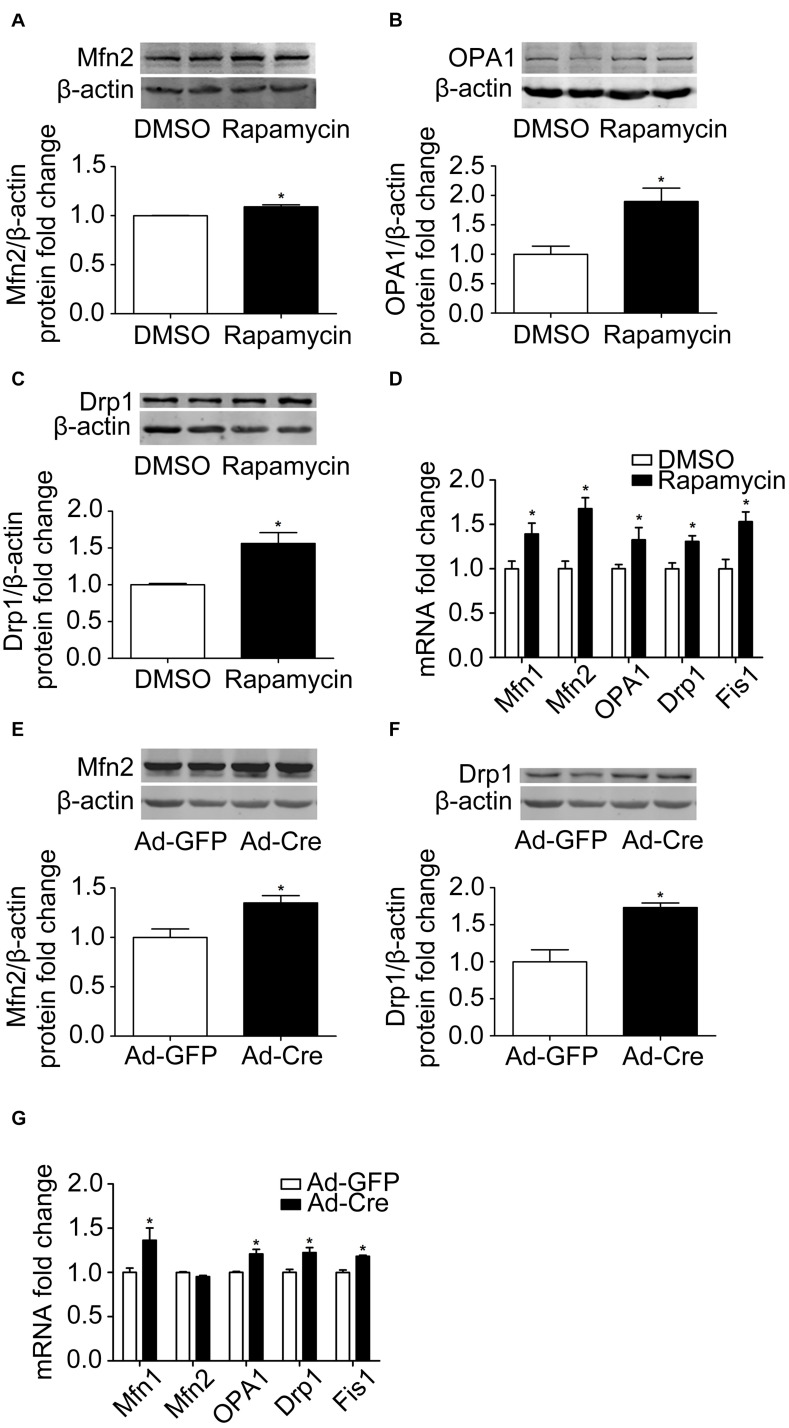
Inhibition of mTOR promotes mitochondrial dynamics of brown adipocytes. **(A–C)** Primary brown adipocytes were isolated from neonatal C57BL/6J mice and cultured for differentiation. Proteins were extracted after 6 days of differentiation. Western blotting of Mfn2, OPA1, and Drp1 was performed with β-actin as a loading control. Relative protein signal intensity was quantified and expressed as means ± SD. **(D)** Total RNAs were extracted after 6 days of differentiation. Real-time PCR was performed to evaluate the expression of mitochondrial dynamics marker genes with β-actin as a loading control and results expressed as means ± SD. *Denotes *P* < 0.05 relative to control. **(E,F)** mTOR^*loxp/loxp*^ brown adipocytes were treated with Cre adenoviruses for 48 h and controls with GFP adenoviruses. RNAs and protein were extracted as described in the methods section and results shown are representative of western blots for Mfn2 and Drp1. β-Actin was used as the loading control. Relative protein signal intensity was quantified and expressed as means ± SD. **(G)** Real-time PCR was performed to evaluate the expression of genes related to mitochondrial dynamics. Levels of mRNA expression were normalized to β-actin and expressed as means ± SD. *P < 0.05 vs. Ad-GFP.

Because activation of mTOR inhibits the mitochondrial dynamics of brown adipocytes, we investigated what would happen if we inhibit the mTOR signaling pathway. Therefore, we used rapamycin for processing. Rapamycin treatment stimulated mitochondrial fusion-related marker Mfn2 and OPA1 (Figures 4A,B) and mitochondrial fission-related marker Drp1 protein expression levels significantly (Figure 4C). The mRNA expression levels of mitochondrial dynamics-related markers, such as Mfn1, Mfn2, OPA1, Drp1, and Fis1, were also significantly increased (Figure 4D).

The same results were also verified in mTOR knockdown cells. As shown in Figures 4E,F, mitochondrial dynamics-related markers Mfn2 and Drp1 protein expression levels were significantly increased. The mRNA expression levels of mitochondrial dynamics-related markers, such as Mfn1, OPA1, Drp1, and Fis1, were also significantly increased (Figure 4G). These results suggest that inhibition of mTOR promotes the mitochondrial dynamics of brown adipocytes.

### mTOR Signaling Pathways Change Mitochondrial Autophagy of Brown Adipocytes

**FIGURE 5:**
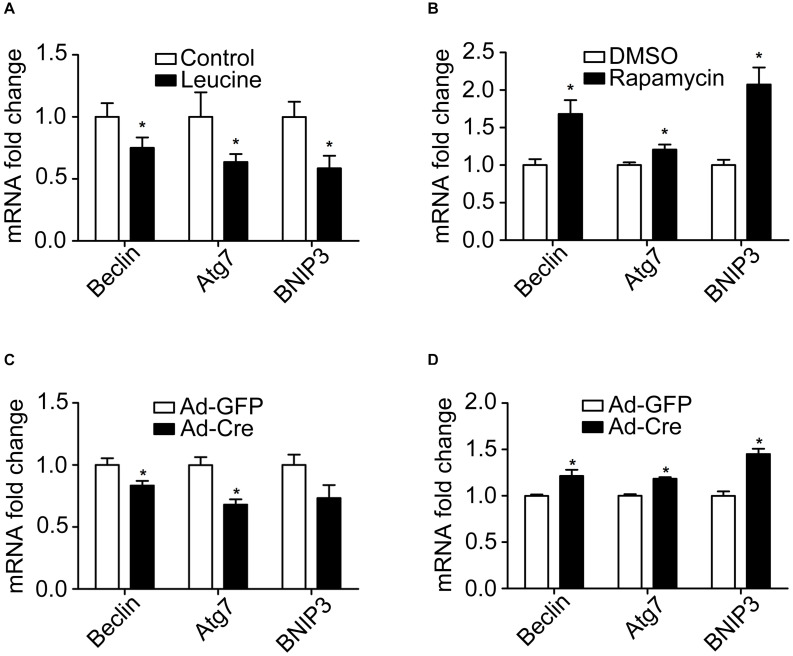
mTOR signaling pathways change mitochondrial autophagy of brown adipocytes. **(A,B)** Primary brown adipocytes were isolated from neonatal C57BL/6J mice and cultured for differentiation. Total RNAs were extracted after 6 days of differentiation. Real-time PCR was performed to evaluate the expression of mitochondrial autophagy marker genes with β-actin as a loading control and results expressed as means ± SD. *Denotes *P* < 0.05 relative to control. **(C)** TSC1^*loxp/loxp*^ brown adipocytes were treated with Cre adenoviruses for 48 h and controls with GFP adenoviruses. RNAs were extracted as described in the methods section. Real-time PCR was performed to evaluate the expression of genes related to mitochondrial autophagy. Levels of mRNA expression were normalized to β-actin and expressed as means ± SD. **(D)** mTOR^*loxp/loxp*^ brown adipocytes were treated with Cre adenoviruses for 48 h and controls with GFP adenoviruses. RNAs were extracted as described in the methods section. Real-time PCR was performed to evaluate the expression of genes related to mitochondrial dynamics. Levels of mRNA expression were normalized to β-actin and expressed as means ± SD. *P < 0.05 vs. Ad-GFP.

Because autophagy plays a key role in the mitochondrial quality control, we investigated the effect of the mTOR signaling pathway on mitochondrial autophagy. We found that leucine treatment significantly decreased the mRNA expression levels of mitochondrial autophagy-related markers, such as Beclin, Atg7, and BNIP3 (Figure 5A), while rapamycin treatment significantly increased the mRNA expression levels of mitochondrial autophagy-related markers (Figure 5B).

Similar results have also been verified in the gene knockdown cells. The mRNA expression levels of mitochondrial autophagy-related markers were significantly decreased in TSC1 knockdown cells (Figure 5C) while significantly they were increased in mTOR knockdown cells (Figure 5D). These results suggest that activation of the mTOR signaling pathway inhibits mitochondrial autophagy, while inhibition of mTOR promotes the mitochondrial autophagy of brown adipocytes.

## Discussion

**FIGURE 6:**
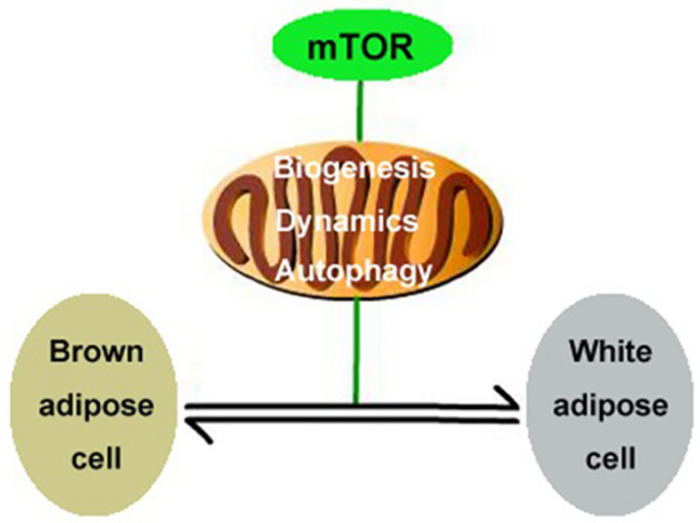
Illustration of putative signaling pathways. mTOR signaling pathway regulates mitochondrial biogenesis, mitochondrial dynamics, and mitophagy in brown adipocytes and therefore plays an important role in modulating the transformation of the adipocyte phenotype.

In the present study, we report that the mTOR signaling pathway regulates mitochondrial biogenesis, mitochondrial dynamics, and mitophagy in brown adipocytes and therefore plays an important role in modulating the transformation of the adipocyte phenotype by regulating mitochondrial quality control (Figure 6). This conclusion is supported by the following observations: (1) Activation of mTOR inhibits mitochondrial biogenesis in brown adipocytes; (2) inhibition of mTOR promotes mitochondrial biogenesis in brown adipocytes; (3) activation of mTOR inhibits mitochondrial dynamics in brown adipocytes; (4) inhibition of mTOR promotes mitochondrial dynamics in brown adipocytes; and (5) the mTOR signaling pathway alters mitochondrial autophagy in brown adipocytes.

The incidence of metabolic diseases such as obesity and type 2 diabetes is increasing. Skeletal muscle, which is rich in mitochondria, is especially prone to metabolic dysfunction and IR (DeFronzo and Tripathy, 2009) as well as reduced glucose uptake, abnormal protein conversion, lipid metabolism disorders, and mitochondrial dysfunction (Koves et al., 2008; Anderson et al., 2009; Nilsson et al., 2010, 2013; Greene et al., 2014). In the case of obesity and type 2 diabetes, skeletal muscle shows changed morphology, damaged quality, and dysfunction of the mitochondria (Kelley et al., 2002; Yokota et al., 2009; Laker et al., 2014) and increased release of reactive oxygen species and lipid by-products (Koves et al., 2008; Klotz et al., 2015). Therefore, mitochondrial quality control and function are closely related to metabolic disease.

Adipose tissue, another important metabolic-related tissue, plays an important role in the pathogenesis and development of obesity and type 2 diabetes. Similar to skeletal muscle cells, brown adipocytes are rich in mitochondria, and as an important factor in thermogenesis, UCP1 is located on the mitochondrial membrane. Mitochondrial function plays an important role in the function of brown adipocytes; therefore, identifying possible disorders in mitochondrial quality is very important to maintain healthy cells. Obesity and type 2 diabetes are associated with damaged mitochondrial quality, and normal physical activity is associated with enhanced mitochondrial activity. Existing studies have shown that mitochondrial quality deterioration caused by a high-fat diet can be improved through physical exercise (Laker et al., 2014). It is also reported that physical activity can promote the expression and secretion of mitochondrial biogenesis-related molecules (Akimoto et al., 2005; Pogozelski et al., 2009; Greene et al., 2012, 2014), mitochondrial fusion-related molecules (Ding et al., 2010; Perry et al., 2010), mitochondrial fission-related molecules (Perry et al., 2010), and mitochondrial autophagy-related molecules (He et al., 2012; Lira et al., 2013). Quality and function of mitochondria in BAT play a vital role in body energy metabolism and energy consumption.

Cellular energy-sensing molecule mTOR is a highly conserved serine-threonine kinase. Our previous study has shown that mTORC1 is activated in obesity, activation of mTORC1 signaling pathway stimulates the transformation from brown adipocytes to white adipocytes, and the application of mTORC1 specificity inhibitor rapamycin can reverse the phenotype transformation (Xiang et al., 2015). In the present study, through mTOR activator leucine processing or specific knockdown of mTOR upstream inhibitory molecule TSC1, mitochondrial biogenesis, dynamics function, and autophagy are significantly reduced in brown adipocytes; inhibition of mTOR significantly enhanced mitochondrial biogenesis, dynamics function, and autophagy in brown adipocytes. However, in our experiments, mitochondrial respiratory capacity and mitochondrial membrane potential did not change significantly after alteration of mTOR activity (Supplementary Figures 2, 3), which need further discussion.

As one of the important functional organelles, mitochondria are abundant in brown adipocytes. Changes in mitochondrial quality and function upon activation or inhibition of mTOR are likely to be the main cause of brown adipocyte/white adipocyte transformation. In other words, in brown adipocytes, with the activation of mTOR there is inhibition of mitochondrial biogenesis, reduction of mitochondrial fusion–fission function, and inhibition of mitochondrial autophagy that in turn induces the conversion of brown adipocytes to white adipocytes. When mTOR signaling was inhibited, enhanced mitochondrial biogenesis, enhanced mitochondrial fusion–fission function, and increased mitochondrial autophagy occurred. This process could inhibit the conversion of brown adipocytes to white adipocytes, reverse this process, or even cause brown or beige phenotypic transformation of white adipocytes. This finding suggests that we can use mTOR as a target for obesity treatment, and by blocking the activity of mTOR, we can change the quality and function of mitochondria, thus promoting the conversion of WAT to BAT and increasing energy expenditure, which may be important for the prevention and treatment of obesity (Xiang et al., 2015; Dai et al., 2018; Shen et al., 2018).

In summary, the mTOR signaling pathway plays an important role in modulating the transformation of adipocyte phenotype by regulating mitochondrial quality control.

## Data Availability Statement

The original contributions presented in the study are included in the article/Supplementary Material, further inquiries can be directed to the corresponding author/s.

## Ethics Statement

The animal study was reviewed and approved by Animal Care and Use Committee of Peking University.

## Author Contributions

BH, WY, LL, YJ, XX, and YL: data collection. BH and YL: writing—original draft preparation. JH, WZ, and YL: writing—review and editing. YL: project administration and funding acquisition. All authors contributed to the article and approved the submitted version.

## Conflict of Interest

The authors declare that the research was conducted in the absence of any commercial or financial relationships that could be construed as a potential conflict of interest.
