# Supplementary material for: Mammalian Target of Rapamycin Signaling Pathway Regulates Mitochondrial Quality Control of Brown Adipocytes in Mice
Source: Front Physiol. 2021 Jul 14;12:638352. doi: 10.3389/fphys.2021.638352 (PMC8317026; doi:10.3389/fphys.2021.638352)
Supplement: Supplementary file 1 [file Data_Sheet_1.DOCX]

**Supplementary figure legends**

**Supplementary Figure 1.** Effective differentiation of brown adipocytes. (A, B) Total RNAs were extracted from primary brown adipocytes after 6 days of differentiation, Real-time PCR was performed to evaluate the expression of brown adipocyte marker genes with β-actin was used as a loading control, and results expressed as means ± SD. (C) Proteins were extracted from primary brown adipocytes after 6 days of differentiation, western blotting of PGC1α and UCP1 were performed with β-actin as a loading control, and relative protein signal intensity was quantified and expressed as means ± SD. (D) Oil red O staining was performed to demonstrate the lipid droplets. *denotes P < 0.05 relative to undifferentiated controls.

**Supplementary Figure 2.** Mitochondrial respiration assay. Primary brown adipocytes were isolated from neonatal C57BL/6J mice and cultured for differentiation. (A&B) Cells were treated with 15 mmol/L of leucine. (C&D) Cells were treated with 1 ng/ml of rapamycin. O_2_ consumption rate (OCR) and respiratory control ratio (RCR) were detected.

**Supplementary Figure 3.** (A) Mitochondrial morphology and (B) mitochondrial membrane potential in brown adipocytes. Primary brown adipocytes were isolated from neonatal C57BL/6J mice and cultured for differentiation. 15 mmol/L of leucine. or 1 ng/ml of rapamycin were added to affect mTOR signaling pathway.
