# Supplementary figures and images for: Mammalian Target of Rapamycin Signaling Pathway Regulates Mitochondrial Quality Control of Brown Adipocytes in Mice
Source: Front Physiol. 2021 Jul 14;12:638352. doi: 10.3389/fphys.2021.638352 (PMC8317026; doi:10.3389/fphys.2021.638352)

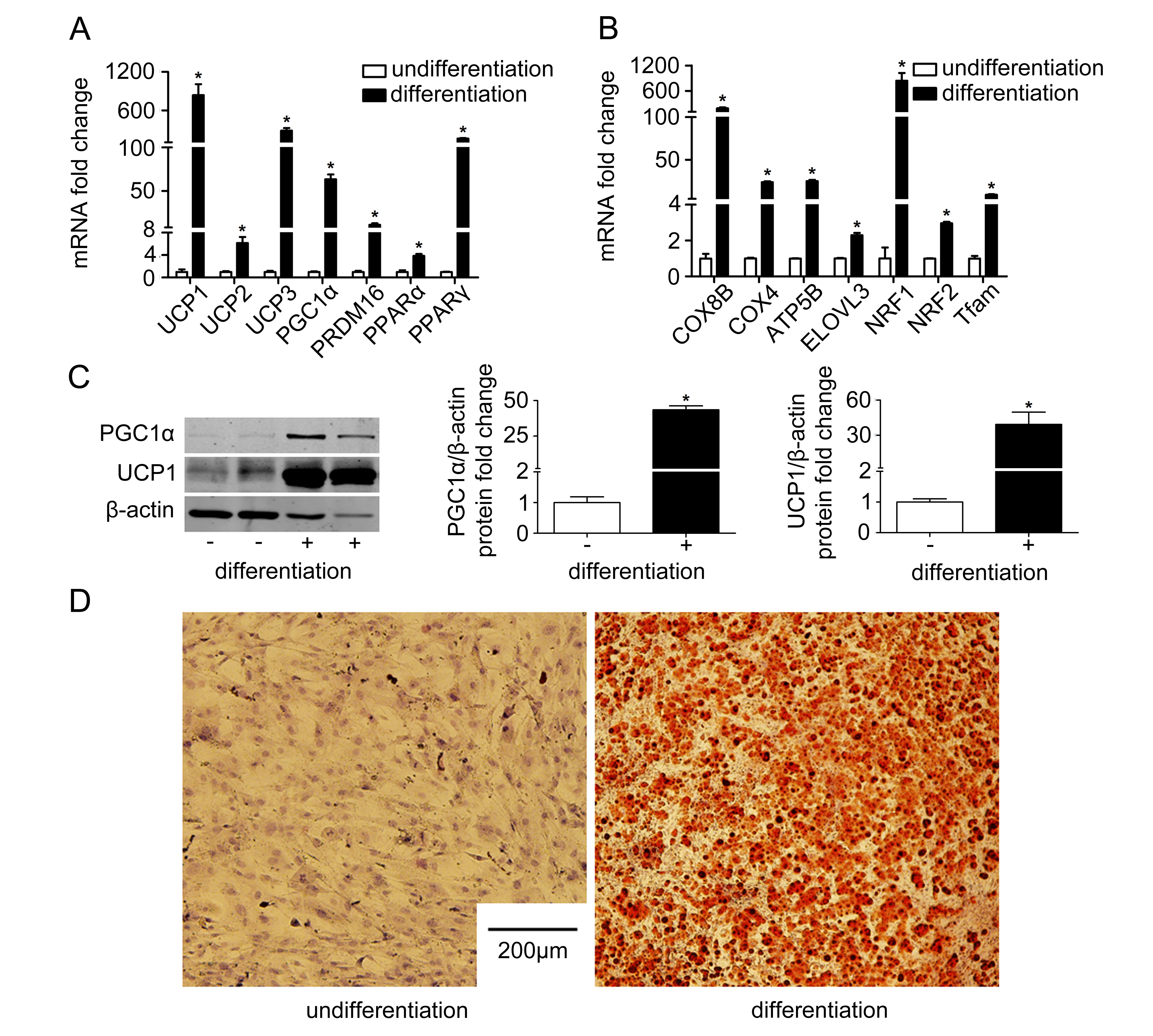

Supplement: Supplementary file 2 [file Image_1.JPEG]

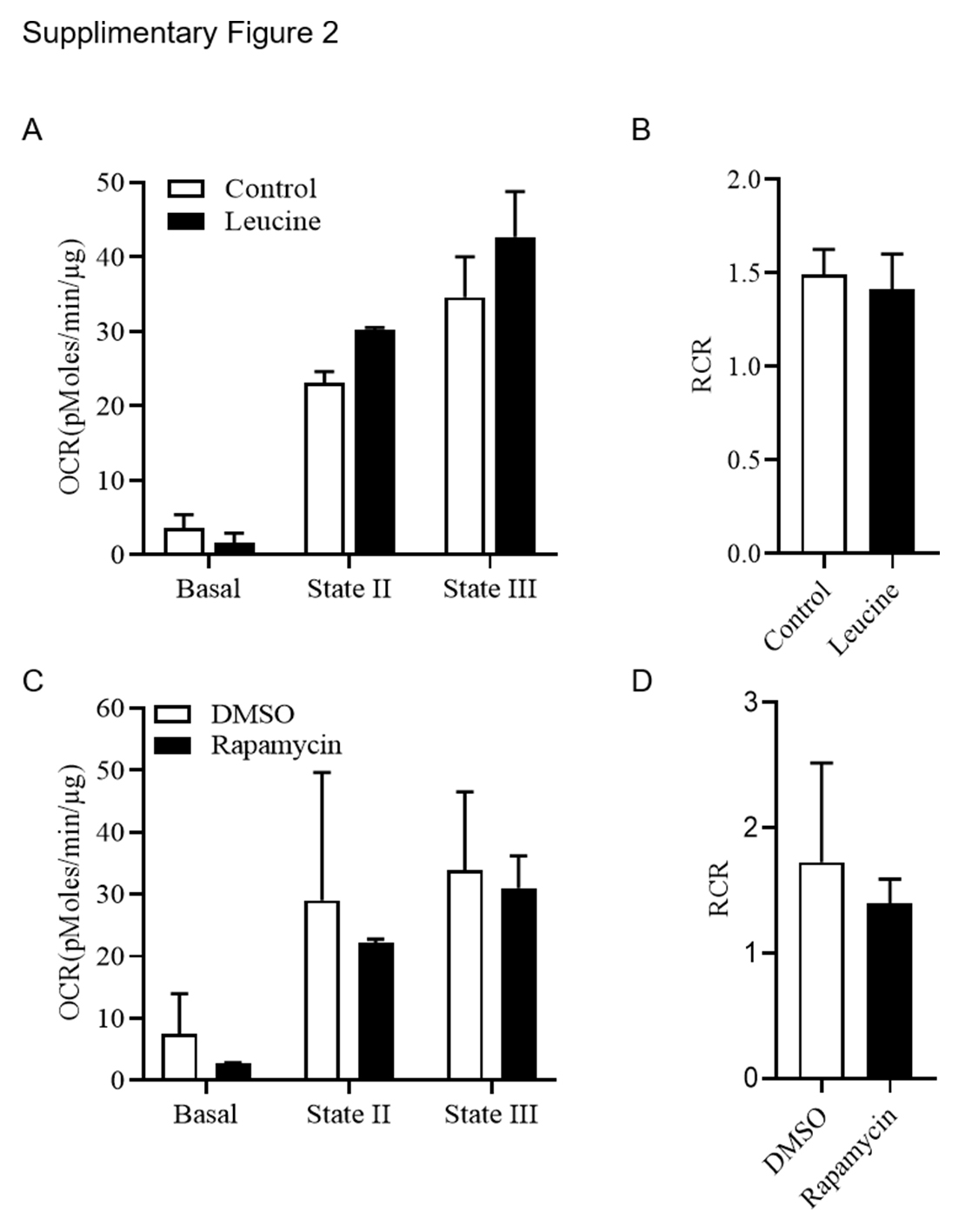

Supplement: Supplementary file 3 [file Image_2.JPEG]

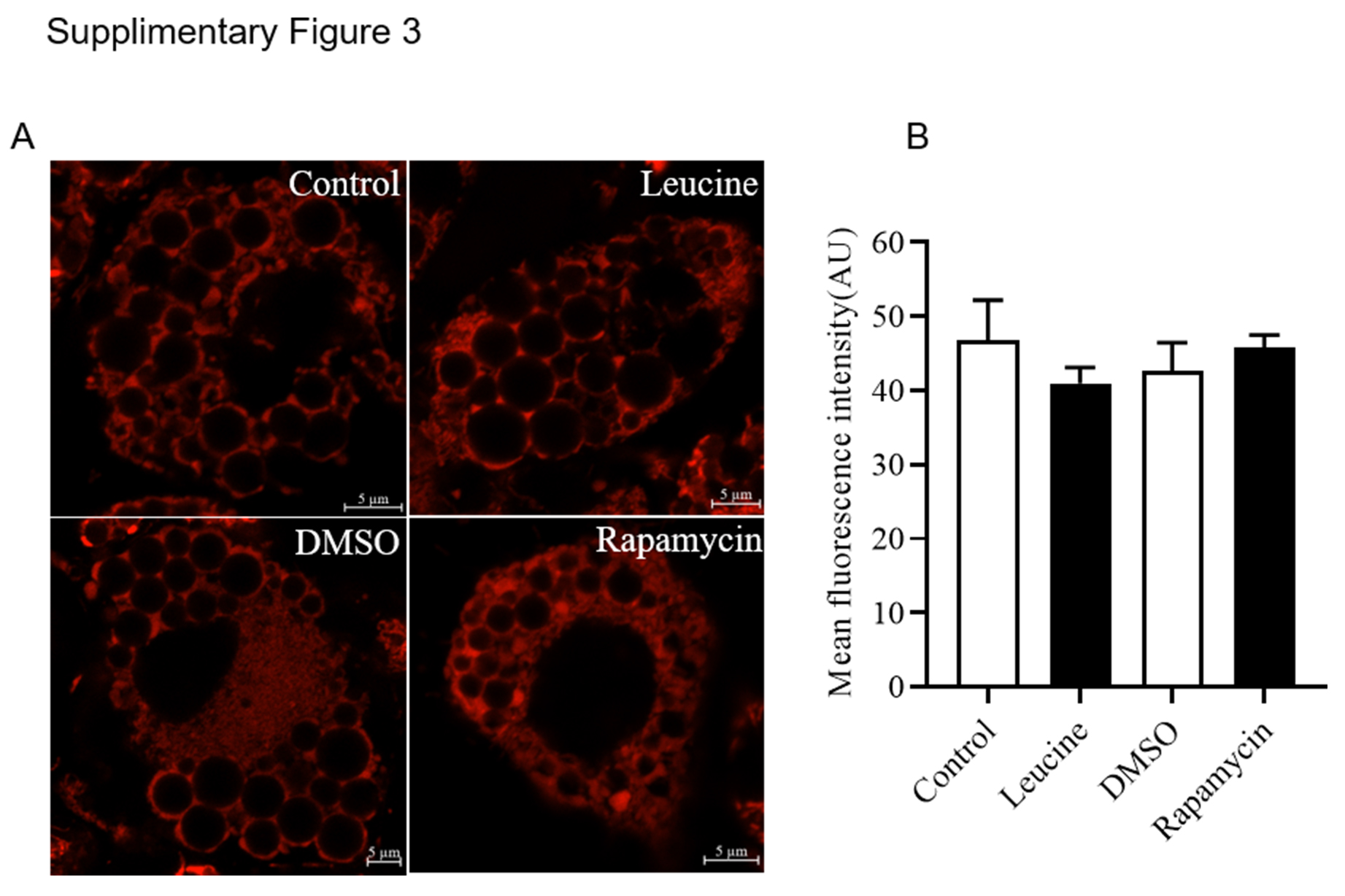

Supplement: Supplementary file 4 [file Image_3.JPEG]
